# Sparse Power-Law Network Model for Reliable Statistical Predictions Based on Sampled Data

**DOI:** 10.3390/e20040257

**Published:** 2018-04-07

**Authors:** Alexander P. Kartun-Giles, Dmitri Krioukov, James P. Gleeson, Yamir Moreno, Ginestra Bianconi

**Affiliations:** 1 School of Mathematical Sciences, Queen Mary University of London, London E1 4NS, UK; 2 Departments of Physics, Mathematics, and Electrical & Computer Engineering, Northeastern University, Boston 02120, MA, USA; 3 MACSI, Department of Mathematics and Statistics, University of Limerick, Limerick V94 T9PX, Ireland; 4 Institute for Biocomputation and Physics of Complex Systems (BIFI), University of Zaragoza, Zaragoza 50013, Spain; 5 Department of Theoretical Physics, Faculty of Sciences, University of Zaragoza, Zaragoza 50013, Spain; 6 Institute for Scientific Interchange (ISI Foundation), Turin 10121, Italy; 7 Complexity Science Hub Vienna, Vienna 22180, Austria

**Keywords:** networks models, projectivity and exchangeability, network entropy, information theory of networks

## Abstract

A projective network model is a model that enables predictions to be made based on a subsample of the network data, with the predictions remaining unchanged if a larger sample is taken into consideration. An exchangeable model is a model that does not depend on the order in which nodes are sampled. Despite a large variety of non-equilibrium (growing) and equilibrium (static) sparse complex network models that are widely used in network science, how to reconcile sparseness (constant average degree) with the desired statistical properties of projectivity and exchangeability is currently an outstanding scientific problem. Here we propose a network process with hidden variables which is projective and can generate sparse power-law networks. Despite the model not being exchangeable, it can be closely related to exchangeable uncorrelated networks as indicated by its information theory characterization and its network entropy. The use of the proposed network process as a null model is here tested on real data, indicating that the model offers a promising avenue for statistical network modelling.

## 1. Introduction

Network science [1,2,3,4] is one of the most rapidly advancing scientific fields of investigation. The success of this field is deeply rooted in its interdisciplinarity. In fact, network science characterizes the underlying structure and dynamics of complex systems ranging from on-line social networks to molecular networks and the brain. Additionally, the theoretical tools and techniques used by network science are coming from different disciplines including statistical mechanics, statistics, machine learning and computer science.

In the last twenty years significant attention has been addressed to modelling framework of complex networks. Since most real networks, from the Internet to molecular networks, are sparse, i.e., they have an average degree that does not depend on the network size, statistical mechanics models focus on modelling sparse networks. These statistical mechanics models can be divided between non-equilibrium growing network models [5,6,7,8,9,10,11,12,13] such as the famous Barabási–Albert model [5] and equilibrium models such as maximum entropy network ensembles [14,15,16,17,18,19] including Exponential Random Networks [16,17,20,21,22] and block models [23,24]. The non-equilibrium growing network models have the power to explain the fundamental mechanisms giving rise to emergent properties such as scale-free distributions [5,6,7,8], degree correlations [6], communities [9,10,11] and network geometry [11,12,13]. On the contrary, maximum network ensembles constitute the least biased models satisfying a given set of constraints. These models are not explanatory but constitute the ideal null hypothesis to which real networks can be compared.

Recently the need to formulate reliable statistical models is receiving significant attention [25]. A reliable statistical model will include projectivity and exchangeability [26,27,28,29,30]. The projectivity of the statistical network model guarantees that the conclusions reached by considering a subsample of the data are consistent with the ones that can be drawn starting from a larger sample of the data. The exchangeability of the nodes implies that the probability of a network does not depend on the specific labels of the nodes. However, how to reconcile these statistical requirements with the sparseness of the networks, i.e., a average degree that is independent of the network size, constitutes a major impasse of network modelling. For instance it has been shown that random uncorrelated networks are only projective if the average degree 〈k〉 of the network increases linearly with the network size *N*, i.e., if the network is maximally dense and 〈k〉=O(N) [27,30,31].

In physical terms, the desired projective and exchangeable network process mimicking the subsequent sampling of an increasing portion of the network is a modelling framework that goes beyond the traditional statistical mechanics division between equilibrium and non-equilibrium network modelling approaches. This observation reinforces the belief that actually combining these two properties might be not an easy task.

Already several works have addressed this problem [32,33,34,35,36,37,38], using different approaches such as relaxing the condition 〈k〉=O(N) but always characterizing models with average degree diverging with the network size *N*, considering edge exchangeable models or alternatively using an embedding space as a basic mechanism to combine sparsity with projectivity and exchangeability [31,39].

Here we propose a network process describing a network evolution mimicking the sampling of a network by subsequently expanding the nodes set. Each node is assigned an hidden variable from a hidden variable distribution. This distribution is the key quantity determining the properties of the network process. If the hidden variable is power-law distributed and the network is sufficiently sparse, the degree distribution displays a power-law tail with the same power-law exponent as the hidden variable distribution.

This model is a projective network process but it is not exchangeable. Nevertheless, this non-equilibrium network model can be directly related to an equilibrium uncorrelated network ensemble in the sparse regime. In fact, by permuting the order in which nodes are sampled it is possible to calculate the probability that two nodes are connected given their corresponding hidden variables. This connection probability is equal to the connection probability in an uncorrelated exchangeable network ensemble in which the hidden variable of each node is identified with half of its expected degree. The “proximity” between the network process and the uncorrelated network ensemble is here quantified by using information theory tools and comparing the entropy of the two models. In particular, we use the entropy of the two network models [14,15,16,17,40] to evaluate the difference in the information content of the two models, finding that the two models have small relative entropy difference.

Finally we study how well the proposed model can be used as a null model for real power-law network datasets. To this end we identify the hidden variable of each node with half of its observed degree and we run the model by adding the nodes in the network according to a random permutation of the nodes’ labels. The degree distribution of the real dataset and the degree distribution of the simulation results are in good agreement when starting from power-law networks, and the agreement remains good if the network is grown by only considering a subsample of the nodes of the real data. We also compare the correlations of the real dataset with the correlations of the simulation results to show that the simulations are able to generate only weak correlations of the degrees. Therefore a more refined model should be formulated to capture this additional network property.

The paper is structured as described in the following. In Section 2 we introduce the definition of the desired statistical properties of network models: *projectivity* and *exchangeability*. In Section 3 we discuss major examples of sparse network models (the Barabási–Albert model and the uncorrelated network ensembles) and characterize them with respect to the properties of projectivity and exchangeability. In Section 4 we present an account of the difficulties in combining projectivity and exchangeability with the sparseness of networks and we give a brief review of the approaches investigated in the recent literature on the subject. In Section 5 we present a network process mimicking a network sampling process. We characterize its structural and dynamical properties relating this non-equilibrum model to equilibrium uncorrelated network ensembles, and we characterize its statistical properties. In Section 6 we show the possible use of the proposed network process as a null model for modelling real power-law network datasets. Finally in Section 7 we give the conclusions.

## 2. Statistical Terms

Projectivity and exchangeability are two very basic and very natural statistical requirements for reliable statistical network models. In physical terms, projectivity is directly related to the principle of locality, while exchangeability is related to symmetry. In this section, we first discuss projectivity and exchangebility to make clear that they really are “must-haves” in any statistically useful network model, while in the next two sections we will comment on difficulties in combining them both in models of sparse networks, i.e., having average degree independent of the network size *N* [41]. While projectivity and exchangeability are desired properties of statistically reliable network models, the relevance and of these requirements for any realistic network model is a subject of scientific debate (see for instance contribution of Karthik Bharath in the discussion of the F. Caron and E. Fox paper [33]). In fact it is often observed that most real networks can hardly be exchangeable. Indeed, in a vast majority of real networks nodes are labelled with labels related to some rich metadata and a random permutation of the nodes labels would result in a different network whose probability to be produced by the same stochastic process that produces the real network is certainly not expected to be equal to the probability with which it generates the real network.

In order to investigate the properties of reliable statistical models we consider a network process mimicking the subsequent sampling a network by expanding the set of sampled nodes and detecting all the interactions among this set of nodes.

To this end we consider a set of networks ${\left\{G_{t}\right\}}_{t\;=\;1,\;2,\;\ldots }$ with $G_{t}=\left(V_{t},E_{t}\right)$ and increasing network size $N_{t}=\left|V_{t}\right|=t$. The sequence of networks defines a network process, i.e., $G_{t}=\left(V_{t},E_{t}\right)$ is an induced subgraph of the network $G_{t^{\prime }}=\left(V_{t^{\prime }},E_{t^{\prime }}\right)$ for all $t<t^{\prime }$ with node set $V_{t}\subset V_{t^{\prime }}$ if $t<t^{\prime }$. We label the nodes in order of their appearance in the network such that

(1) $$\begin{array}{c} V_{t}=\left\{1,2,\ldots ,t\right\}. \end{array}$$

and assign a probability $P(G_{t})$ to each network $G_{t}$.

### 2.1. Projectivity

Given the set of networks ${\left\{G_{t}\right\}}_{t\;=\;1,\;2,\;\ldots }$*projectivity* implies that the statistical properties of the network $G_{t}$ are directly related to the statistical properties of the network $G_{t^{\prime }}$ with $t^{\prime }>t$ by a proper marginalization of the probability of the network $G_{t^{\prime }}$ over its subgraph $G_{t}$.

By definition [26,27], a projective network model is a model that attributes a given probability $P(G_{t})$ to each network $G_{t}$ of the sequence, such that

$$P\left(\pi_{t^{\prime },t}\left(G_{t^{\prime }}\right)\right)=P\left(G_{t}\right),$$

where the projective map $\pi_{t^{\prime },t}$ maps networks $G_{t^{\prime }}$ of a larger size $t^{\prime }>t$ to their subgraph $G_{t}$ of a smaller size *t*.

In other words this means that one can first generate a larger graph $G_{t^{\prime }}$ using the model, then reduce its size to *t* by throwing out some $t^{\prime }-t$ nodes according to the projective map specification, and the probability with which the resulting graph $G_{t}$ is generated using this two-step procedure will be the same as if graph $G_{t}$ was generated by the model directly.

### 2.2. Exchangeability

Exchangeability implies that the order in which two nodes are observed or labelled is not important. Specifically, a network model is exchangeable if, by definition [29,30], the probability $P(G_{t})$ of a network $G_{t}=\left(V_{t},E_{t}\right)$ is independent on the nodes labels, i.e.,

(2) $$\begin{array}{c} P\left(G_{t}\right)=P\left({\tilde{G}}_{t}\right) \end{array}$$

where ${\tilde{G}}_{t}$ is any network isomorphic to the network $G_{t}$, i.e., it is any network obtained from the network $G_{t}$ by permuting the nodes labels ${\left\{i\right\}}_{i\;=\;1,\;2,\;\ldots ,\;N}$ according to the permutation σ. If a network model is exchangeable it follows that the marginal the probability $p_{ij}$ of the generic link between node *i* and node *j* is unchanged if the node labels are permuted, implying that they are sampled in a different order, i.e.,

(3) $$\begin{array}{c} p_{ij}=p_{\sigma (i),\sigma (j)}. \end{array}$$

Therefore exchangeability enforces the *symmetry* of the model with respect to the group of graph isomorphisms.

## 3. Characterization of Relevant Sparse Network Models from the Statistical Perspective

In this section we investigate major examples of non-equilibrium (growing) network models and equilibrium (static) network models widely used to model sparse complex networks. In particular we discuss the Barabási–Albert model [5] and the uncorrelated network ensembles from the statistical perspective. This discussion will reveal that neither of these two very popular frameworks for modelling sparse complex networks display both projectivity and exchangeability, indicating the difficulties in combining these properties with the sparseness of the networks.

### 3.1. Barabási–Albert Model

The Barabási–Albert model begins with an initial finite network and at each time *t* a new node enters in the network and is connected to the network by establishing *m* new links. Each of these links connect the new node to a node *i* with degree $k_{i}$ chosen with probability

(4) $$\begin{array}{c} {\tilde{\Pi }}_{i}=\frac{k_{i}}{\sum_{i^{\prime }}k_{i^{\prime }}}. \end{array}$$

This probability enforces *preferential attachment*, i.e., allows nodes with higher degree to more rapidly acquire new links.

The Barabási–Albert model describes a model that is projective, because as the network grows the network $G_{t}$ obtained at time *t* is an induced subgraph of the network $G_{t^{\prime }}$ obtained at a later time $t^{\prime }>t$. However the Barabási–Albert model is not exchangeable. The fact that the network is not exchangeable is revealed for instance by the expression for the average number of links $k_{i}\left(t,t_{i}\right)$ of a node *i* arrived in the network at time $t_{i}$,

(5) $$\begin{array}{c} k_{i}\left(t,t_{i}\right)=m{\left(\frac{t}{t_{i}}\right)}^{1/2}. \end{array}$$

This expression explicitly indicates that the older nodes are statistically different from the younger nodes, and their degree is much larger than that of younger nodes. Additionally it is possible to observe that the model is not exchangeable because the order of the addition of the nodes, i.e., their time of arrival in the network, is the key property that determines the connection probability [42], i.e.,

(6) $$\begin{array}{c} p_{ij}≃\frac{m}{2}\frac{1}{\sqrt{t_{i}t_{j}}}. \end{array}$$

Nevertheless we observe the interesting property that for this model the connection probability $p_{ij}$ between node *i* and node *j* can be also expressed as

(7) $$\begin{array}{c} p_{ij}≃\frac{k_{i}\left(t,t_{i}\right)k_{j}\left(t,t_{j}\right)}{\sum_{i^{\prime }}k_{i^{\prime }}\left(t,t_{i^{\prime }}\right)}, \end{array}$$

indicating that actually, although the network process has different statistical properties than the uncorrelated network with the same degree distribution, the expected degree correlations are weak. The relation between the Barabási–Albert (BA) model and the uncorrelated network ensemble with the same degree distribution is investigated in detail using information theoretic tools in Ref. [43].

### 3.2. Uncorrelated Network Ensembles

The Barabási–Albert model is projective but not exchangeable. On the contrary the widely used uncorrelated network ensembles are exchangeable models but they are not projective in the sparse regime. In order to show this let us consider an uncorrelated network model in which each node *i* has an expected degree $\theta_{i}$, where the expected degrees of the nodes are consistent with a structural cutoff, i.e.,

(8) $$\begin{array}{c} \theta_{i}\leq \sqrt{\langle \theta \rangle N}. \end{array}$$

In this case the probability $p_{ij}$ of a link between node *i* and node *j* is given by

(9) $$\begin{array}{c} p_{ij}=\frac{\theta_{i}\theta_{j}}{\langle \theta \rangle N}, \end{array}$$

and therefore it only depends on the expected degrees $\theta_{i}$ and $\theta_{j}$ of the nodes *i* and *j* and not on the order in which node *i* and node *j* have been sampled. The model is therefore exchangeable as long as we consider the simultaneous permutation of the node labels and the expected degrees of the nodes. However if we consider a large sample of the network with $N^{\prime }>N$ nodes, we see that the model is projective if and only if it is also dense, with the number of links scaling as $L=O(N^{2})$. In fact if we assume that in the larger sample the expected degrees of nodes *i* and *j* are given by $\theta_{i}^{\prime }$ and $\theta_{j}^{\prime }$, the probability that node *i* and node *j* are connected in the larger network models including $N^{\prime }$ nodes is

(10) $$\begin{array}{c} p_{ij}^{\prime }=\frac{\theta_{i}^{\prime }\theta_{j}^{\prime }}{\langle \theta^{\prime }\rangle N^{\prime }} \end{array}$$

If we impose projectivity, i.e.,

(11) $$\begin{array}{c} p_{ij}=p_{ij}^{\prime } \end{array}$$

for i,j≤N, and we assume that the number of nodes $N^{\prime }>N$ can be written as

(12) $$\begin{array}{c} N^{\prime }=zN, \end{array}$$

it is easy to see that we should also have

(13) $$\begin{array}{cc} \theta_{i}^{\prime } & =z\theta_{i},\\ \langle \theta_{i}^{\prime }\rangle & =z\langle \theta \rangle . \end{array}$$

Therefore to guarantee projectivity the expected degree of each node should grow linearly with the network size, resulting in a dense network with the total number of links *L* scaling with the network size *N* as $L=O(N^{2})$. This implies that the random network G(N,p) with *p* independent of *N* is an exchangeable model whereas the Poisson random network G(N,p) with $p=\frac{z}{N}$ and *z* independent of *N* is not exchangeable. In fact one cannot throw out $N^{\prime }-N$ nodes from a network of size $N^{\prime }$ produced by $G(N^{\prime },z/N^{\prime })$, and hope that the resulting network will have the same probability as in G(N,z/N), simply because the links in the $G(N^{\prime },z/N^{\prime })$ and G(N,z/N) ensembles exist with different probabilities $z/N^{\prime }$ and z/N that depend on the graph size *N*. Alternatively, if one attempts to formulate G(N,z/N) as a growing model, then since the edge existence probability depends on *N*, the addition of a new node affects the probability of existence of edges in the existing network. Since this probability is a decreasing function of *N* (z/N), upon the addition of a new node all the existing edges must be removed with some probability (1/N). In other words, in such a growing model new node additions must necessarily affect the existing network structure.

## 4. Impasse with Sparsity

Surprisingly, combining projectivity and exchangeability with the additional constraint of sparsity, i.e., the requirement that the average degree of the sampled networks is independent of the network size, has been a major impasse. If we exclude spatially embedded networks [31], to the best of our knowledge there exists no model of sparse networks that would be both projective and exchangeable at the same time. This situation is in stark contrast with the case of dense graphs. Dense graphs are known to have well-defined thermodynamic limits known as graphons, and any graphon-based network model is both exchangeable and projective [30].

The thermodynamic limits of sparse graphs are at present quite poorly understood, which appears to be one of the reasons behind the mentioned impasse. Several attempts have been made to understand the limits of sparse graphs, including, for example, sparse $L^{p}$ graphons [32], which are not projective, or stretched graphons a.k.a. graphexes [33,34,35]. In the latter case, graphs are sparse, exchangeable and projective, but with two major caveats:

(1) the average degree cannot be constant, it must diverge with *N* (but possibly slower than linearly),
(2) exchangeability is completely redefined: it is not with respect to node labels 1,…,N, but with respect to artificial labels which are positive real numbers.

Another class of attempts suggests to completely give up on the node label exchangeability requirement, and to consider edge exchangeability instead, e.g., using variations of Pitman–Yor processes [36,37,38]. It remains unclear at present whether these developments imply that too many network models that were found to be quite useful in practice and that do use node labels 1,…,N, are statistically hopeless. It seems more likely that further research is needed to understand and resolve this projectivity vs. exchangeability impasse in sparse network models.

### Proposed Solution of the Impasse Based on Network Geometry

In [31] it was shown that a generic network model is projective if the probability of edge existence, i.e., the connection probability, does not depend on the network size *N*. In fact if the connection probability does depend on *N*, then, the addition of new nodes to the existing network in the growing formulation of the model necessarily affects the existing network structure and the network cannot be projective.

In order to formulate network models in which the connection probability does not depend on the network size *N*, embedding networks in space can turn out to be very useful. In fact spatially embedded networks can combine projectivity with a constant average degree [31] as their spatial embedding ensures projectivity when the connection probability is *local* and nodes connect typically to nodes that are spatially close. For instance if the nodes are uniformly distributed in $R^{2}$ and each node connects only to the nodes with a constant radius $r_{0}$, by sampling the network by progressively expanding the spatial region of interest we can build a projective model with constant average degree. This is clearly a realistic scenario in most real networks as it unlikely that a local event in a spatial network causes a global change in the network. For instance in the Internet, the appearance of a new customer of a local Internet provider in Bolivia cannot lead to immediate severance of customers by a local Internet provider in Bhutan.

It turns out that models that are not explicitly constructed from spatial embeddings can also be analysed using geometrical arguments, hence shedding light on their statistical properties. In this vein, it was recently shown that the hypersoft configuration model, which defines maximum-entropy random graphs with a given degree distribution, is sparse and *either* exchangeable *or* projective [39]. Both sparsity and exchangeability definitions are traditional in the model, i.e., the average degree is constant and exchangeability is with respect to labels 1,…,N, so that the only caveats are in “either-or” and also in that this “either-or” is achieved only for specific degree distributions (power law with exponent γ=3 in [39]).

In the *exchangeable* equilibrium formulation of the model, nodes are points sprinkled at random onto an interval $A_{N}$ of an *N*-dependent length $L_{N}$, where $L_{N}$ is a growing function of *N*, according to a non-uniform point density (if this point density is exponential, then the resulting degree distribution is a power law), and then all pairs of points/nodes *i* and *j*, j>i=1,…,N, at sprinkled coordinates $x_{i}$ and $x_{j}$ are connected by an edge with the entropy-maximizing Fermi–Dirac connection probability

(14) $$\begin{array}{c} p\left(x_{i},x_{j}\right)=\frac{1}{e^{x_{i}+x_{j}}+1} \end{array}$$

that does not depend on the network size *N*.

In the *projective* growing formulation of the same model, the interval $A_{N}$ grows with *N*, its length growing according to $L_{n}$, new node N+1 appears in the interval increment $A_{N+1}\backslash A_{N}$ of length $L_{N+1}-L_{N}$, and then connects to existing nodes with the same connection probability as in the exchangeable formulation.

The difficulty of combining projectivity and exchangeability is evident in this example: in the exchangeable formulation, node labels *i* are random and uncorrelated with their coordinates $x_{i}$, while in the projective formulation, nodes are labelled in the increasing order of their coordinates: $i<j\Leftrightarrow x_{i}<x_{j}$. If nodes are labelled this way, then the projective map $\pi_{N^{\prime },N}$ trivially throws out nodes with labels $N+1,\ldots ,N^{\prime }$, and the resulting graph satisfies the projectivity requirement since the connection probability does not depend on *N*, and since the remaining *N* nodes lie in $A_{N}$. If the node labels are random, however, as they are in the exchangeable formulation, then it remains unclear if even an asymptotically correct projective map can be constructed.

## 5. Statistical Mechanics Model with Hidden Variables

Our goal is here to reconcile sparseness with a reliable statistical modelling framework without assuming the existence of an embedding geometrical space. In this endeavour we will define a projective network process yielding a sequence of networks growing by the subsequent addition of nodes and links. To each node *i* we associate a hidden variable $\theta_{i}$ that is a proxy for the degree that the node will acquire in the model. The statistical properties of the network model when we average over all the possible sequences determining the subsequent addition of the links obey scaling laws and reduce to the uncorrelated network model of any size *N* in the sparse regime.

Although this model does not ultimately reconcile sparseness with both exchageability and projectivity, we will see in Section 6 that it provides a very reliable null model for power-law networks also if only a subsample of the original network is considered.

### 5.1. The Model

The model can be interpreted as a weighted growing network model where we allow multiedges. In the model every node *i* is assigned a hidden variable $\theta_{i}$ from a hidden variable distribution ρ(θ).

Starting at t=1 from a single isolated node, at each time t>1 a new node *i* is added to the network and draws $\kappa_{i}$ links to the existing nodes of the network, where $\kappa_{i}$ is chosen according to the Poisson distribution with average $\theta_{i}$, i.e.,

(15) $$\begin{array}{c} \hat{P}\left(\kappa_{i}|\theta_{i}\right)=\frac{1}{\kappa_{i}!}\theta_{i}^{\kappa_{i}}e^{-\theta_{i}}. \end{array}$$

Each new link is attached to a node *j* already present in the network with probability

(16) $$\begin{array}{c} \Pi_{j}=\frac{\theta_{j}}{\sum_{r=1}^{t-1}\theta_{r}}. \end{array}$$

Note that not all the new links will yield new connections because the nodes *i* and *j* might be already connected. Additionally note that this model does not implement preferential attachment as the linking probability is only dependent on the externally attributed hidden variable $\theta_{i}$ and not to the dynamically acquired degree $k_{i}$. Whenever a new link connects node *i* to an already connected node *j* the multi-edge between node *i* and node *j* is reinforced, i.e., the weight of the links between node *i* and node *j* increases by one.

Here and in the following we will indicate by a the adjacency matrix of the network, with $t_{i}$ the time at which node *i* has been added to the network, with $k_{i}$ the node degree and with $s_{i}$ the node strength, i.e., the sum of the weights of the links incident to node *i*.

### 5.2. The Strength of a Node and Its Dependence on the Hidden Variable θ

The hidden variable $\theta_{i}$ modulates the temporal evolution of the strength of the node *i*. In fact in the mean-field approach [1,5,44], since at each time an average of 〈θ〉 links are added and reinforced, the average strength $s_{i}\left(t|t_{i},\theta_{i},\kappa_{i}\right)$ of node *i* given the time $t_{i}$ of its arrival in the network, its hidden variable $\theta_{i}$ and its initial strength $\kappa_{i}$ obeys the equation

(17) $$\begin{array}{c} \frac{ds_{i}}{dt}=\langle \theta \rangle \frac{\theta_{i}}{\langle \theta \rangle t}=\frac{\theta_{i}}{t} \end{array}$$

with initial condition $s_{i}\left(t_{i}|t_{i},\theta_{i},\kappa_{i}\right)=\kappa_{i}$. The solution of this equation is

(18) $$\begin{array}{c} s_{i}\left(t|\theta_{i},\kappa_{i}\right)=\theta_{i}\ln\left(\frac{t}{t_{i}}\right)+\kappa_{i}. \end{array}$$

Therefore in this model the strength depends both on the time of arrival of the node in the network and on its hidden variable. If we average the strength over the nodes with the same hidden variable however, we see that the average strength ${\tilde{s}}_{i}\left(\theta_{i}\right)$ of nodes with hidden variable $\theta_{i}$ is given in the large network limit t≫1 by

(19) $$\begin{array}{c} {\tilde{s}}_{i}\left(\theta_{i}\right)=2\theta_{i}. \end{array}$$

In fact we have

(20) $$\begin{array}{ccc} \langle \kappa_{i}|\theta_{i}\rangle & = & \theta_{i}\\ {\tilde{s}}_{i}\left(\theta_{i}\right) & = & \frac{1}{t}\int_{1}^{t}\theta_{i}\ln\left(\frac{t}{t_{i}}\right)dt_{i}+\langle \kappa_{i}|\theta_{i}\rangle \\ & = & 2\theta_{i}+O\left(\frac{\ln t}{t}\right). \end{array}$$

This implies that if we attribute to a node a hidden variable $\theta_{i}$ and we consider a set of models in which the time of arrival of node *i* is taken randomly, the strength of node *i* is (on average over the different network models) determined only by its hidden variable.

### 5.3. Strength Distribution

The strength distribution of the model is a convolution of exponentials. To find the strength distribution we use the master equation approach [44] under the assumption that the hidden variable distribution has a well defined average value 〈θ〉. To this end we write the equation for $N_{\theta }^{t}\left(s\right)$, the average number of nodes with hidden variable θ that have strength s≥0 at time *t*, as

(21) $$\begin{array}{c} \frac{N_{\theta }^{t}\left(s\right)}{dt}=\langle \theta \rangle \Pi \left(\theta \right)N_{\theta }^{t}\left(s-1\right)\left[1-\delta \left(s,0\right)\right]-\langle \theta \rangle \Pi \left(\theta \right)N_{\theta }^{t}\left(s\right)+\rho \left(\theta \right)\hat{P}\left(\kappa =s|\theta \right), \end{array}$$

where δ(x,y) indicates the Kronecker delta and where we denote by Π(θ) the probability that a node with hidden variable θ is attached to the new node arrived in the network at time *t* by one of its connections, i.e.,

(22) $$\begin{array}{c} \Pi \left(\theta \right)=\frac{\theta }{\sum_{\theta^{\prime }}\theta^{\prime }\sum_{s}N_{\theta^{\prime }}^{t}\left(s\right)}≃\frac{\theta }{\langle \theta \rangle t}. \end{array}$$

Given the continuous growth of the network asymptotically in time, for t≫1 it is possible to assume that

(23) $$\begin{array}{c} N_{\theta }^{t}\left(s\right)≃tP_{\theta }\left(s\right), \end{array}$$

where $P_{\theta }\left(s\right)$ is the probability that a random node has strength *s* and hidden variable θ.

By inserting this asymptotic expression in the master Equation (21) and solving for $P_{\theta }\left(s\right)$ we get

(24) $$\begin{array}{c} P_{\theta }\left(s\right)=\rho \left(\theta \right)\sum_{\kappa =0}^{s}\hat{P}\left(\kappa |\theta \right)\frac{1}{1+\theta }{\left(\frac{\theta }{1+\theta }\right)}^{s-\kappa }. \end{array}$$

Therefore given the value of the hidden variable θ and the initial number of links κ the strength distribution is exponential. The overall strength distribution P(s) of the model determining the probability that a random node has strength *s* is given by the integral of $P_{\theta }\left(s\right)$ over all possible value of the hidden variable θ, i.e.,

(25) $$\begin{array}{c} P\left(s\right)=\int d\theta \rho \left(\theta \right)\sum_{\kappa =0}^{s}\hat{P}\left(\kappa |\theta \right)\frac{1}{1+\theta }{\left(\frac{\theta }{1+\theta }\right)}^{s-\kappa }. \end{array}$$

This result reveals that the strength distribution can be different from the distribution of hidden variables. For instance if all the hidden variables are the same, the strength distribution will still allow for fluctuations of the strengths. However for power-law hidden variable distributions

(26) $$\begin{array}{c} \rho \left(\theta \right)≃C\theta^{-\gamma } \end{array}$$

the strength distribution has a power-law tail with the same exponent γ

(27) $$\begin{array}{c} P\left(s\right)≃\hat{C}s^{-\gamma } \end{array}$$

for s≫1. In fact, by inserting the explicit expression of $\hat{P}\left(\kappa |\theta \right)$ and of ρ(θ) in Equation (25) we get

(28) $$\begin{array}{c} P\left(s\right)=C\int d\theta \frac{\theta^{-\gamma }}{1+\theta }\sum_{\kappa =0}^{s}\frac{1}{\kappa !}\frac{\theta^{s}}{{\left(\theta +1\right)}^{s-k}}e^{-\theta }. \end{array}$$

For s≫1 we can approximate the sum over κ with the infinite sum getting

(29) $$\begin{array}{c} P\left(s\right)=C\int d\theta \frac{\theta^{-\gamma }}{1+\theta }{\left(\frac{\theta }{\theta +1}\right)}^{s}e^{-1}≃\hat{C}s^{-\gamma } \end{array}$$

where the last expression is valid if s≫1. Therefore, although in general it is not true that the hidden variable distribution is the same as the strength distribution, in the case of power-law distributed hidden variables the strength distribution displays a power-law tail with the same exponent. Note that this is valid for power-law exponents in the range γ∈(2,3] but also in the range γ∈(1,2]. Therefore in this case the hidden variables can be used to directly tune the strength distribution.

### 5.4. Connection Probability

In this section we derive the expression for the connection probability between any two nodes. Let us consider the probability $P(a_{ij}=1|\theta_{i},\theta_{j},\kappa_{j},t_{j}>t_{i})$ that node *i* is connected to node *j*, i.e., $a_{ij}=1$ given the hidden variables of node *i* and node *j*, their time of arrival with $t_{j}>t_{i}$ and the initial strength $\kappa_{j}$ of node *j*. This probability is one minus the probability that all of the initial links of node *j* do not connect to node *i*, i.e.,

(30) $$\begin{array}{c} P\left(a_{ij}=1|\theta_{i},\theta_{j}\kappa_{j},t_{i}<t_{j}\right)=1-{\left(1-\frac{\theta_{i}}{\sum_{r}\theta_{r}}\right)}^{\kappa_{j}}. \end{array}$$

If we now average over the probability $\hat{P}\left(\kappa_{j}|\theta_{j}\right)$ we get the closed form expression

(31) $$\begin{array}{c} P\left(a_{ij}=1|\theta_{i},\theta_{j},t_{j},t_{i}<t_{j}\right)=\sum_{\kappa_{j}}P\left(\kappa_{j}\right)\left[1-{\left(1-\frac{\theta_{i}}{\sum_{r=1}^{j}\theta_{r}}\right)}^{\kappa_{j}}\right]=\left[1-\exp\left(-\frac{\theta_{i}\theta_{j}}{\langle \theta \rangle t_{j}}\right)\right], \end{array}$$

where we have assumed that the average of the hidden variables $\langle \theta \rangle$ is well defined. Therefore we have found that the connection probability between two nodes depends both on the hidden variables and on their time of arrival in the network. It follows that the model is not expected to be exchangeable, as this would require a connection probability independent of the time of arrival of the two nodes. However the fact that this connection probability does not *only* depend on the time of arrival of the nodes in the network (or the order in which they are sampled) can be a useful characteristic of a reliable statistical model.

### 5.5. Degree Distribution in the Sparse Regime

Here we derive the degree distribution of the model in the sparse regime, when we can assume that $p_{ij}\ll 1$. We will show that in this regime, each node has a Poisson degree distribution with an expected average degree ${\bar{k}}_{i}$ depending both on the value of its hidden variable and on the time of its arrival in the network.

The probability $P(k_{i}|\theta_{i},t_{i})$ that a node *i* arrived in the network at time $t_{i}$ and, having hidden variable $\theta_{i}$, has degree $k_{i}$ can be calculated starting from the connection probabilities $p_{ij}$ given by Equation (31). Let us indicate with $a_{i}=\left\{a_{ij}|j\in \left\{1,2,\ldots ,N\right\}\right\}$ the elements of the adjacency matrix in the *i*-th row indicating the connections of node *i*. Since node *i* is connected with each node *j* with probability $p_{ij}$ given by Equation (31), the probability $P(a_{i})$ is given by

(32) $$\begin{array}{c} P\left(a_{i}\right)=\prod_{j=1}^{N}\left[p_{ij}a_{ij}+\left(1-p_{ij}\right)\left(1-a_{ij}\right)\right]. \end{array}$$

Using this result we can express the probability $P(k_{i}|\theta_{i},t_{i})$ that node *i* has degree $k_{i}$ as

(33) $$\begin{array}{ccc} P(k_{i}|\theta_{i},t_{i}) & = & \sum_{a_{i}}P_{i}\left(a_{i}\right)\delta \left(k_{i},\sum_{j=1}^{N}a_{ij}\right)=\sum_{a_{i}}P\left(a_{i}\right)\int \frac{d\omega }{\sqrt{2\pi }}e^{-i\omega (k_{i}-\sum_{j=1}^{N}a_{ij})} \end{array}$$

where we have used the integral representation of the Kronecker delta δ(x,y). By performing the sum over all the elements of $a_{i}$ we get

(34) $$\begin{array}{ccc} P(k_{i}|\theta_{i},t_{i}) & = & \int \frac{d\omega }{\sqrt{2\pi }}e^{-i\omega k_{i}}\prod_{j=1}^{N}\left[1-p_{ij}\left(1-e^{-i\omega }\right)\right]=\int \frac{d\omega }{\sqrt{2\pi }}e^{F(\omega )} \end{array}$$

where

(35) $$\begin{array}{cc} F(\omega ) & =-i\omega k_{i}+\sum_{j=1}^{N}\ln\left[1-p_{ij}\left(1-e^{-i\omega }\right)\right]. \end{array}$$

For $p_{ij}\ll 1$ we can approximate F(ω) with

(36) $$\begin{array}{ccc} F(\omega ) & = & i\omega k_{i}-\sum_{j=1}^{N}p_{ij}\left(1-e^{-i\omega }\right)=i\omega k_{i}-{\bar{k}}_{i}\left(1-e^{-i\omega }\right) \end{array}$$

where ${\bar{k}}_{i}$ is the expected degree of node *i* given by

(37) $$\begin{array}{c} {\bar{k}}_{i}=\sum_{j=1}^{N}p_{ij}. \end{array}$$

Note here that since the connection probability $p_{ij}$ depends both on the hidden variables of the nodes *i* and *j* and on their arrival time in the network, it follows that also the expected degree ${\bar{k}}_{i}$ of node *i* will be both a function of the node’s hidden variable and its time of arrival in the network. Using Equations (34) and (36) we can derive the explicit expression for $P(k_{i}|\theta_{i},t_{i})$. In fact we have

(38) $$\begin{array}{ccc} P(k_{i}|\theta_{i},t_{i}) & ≃ & \int \frac{d\omega }{\sqrt{2\pi }}e^{i\omega k_{i}-{\bar{k}}_{i}\left(1-e^{-i\omega }\right)}=\sum_{h=0}^{\infty }\frac{1}{h!}{\bar{k}}_{i}^{h}e^{-{\bar{k}}_{i}}\int \frac{d\omega }{\sqrt{2\pi }}e^{i\omega (k_{i}-h)}, \end{array}$$

and by identifying the last integral with the Kronecker delta $\delta (h,k_{i})$ we get the Poisson distribution

(39) $$\begin{array}{c} P\left(k_{i}|\theta_{i},t_{i}\right)=\frac{{\bar{k}}_{i}^{k_{i}}}{k_{i}!}e^{-{\bar{k}}_{i}}. \end{array}$$

Therefore the probability that node *i*, which arrived in the network at time $t_{i}$ with hidden variable $\theta_{i}$, has degree $k_{i}$ is given by the Poisson distribution with average ${\bar{k}}_{i}$ given by Equation (37). It follows that the degree distribution P(k) of the network at time *t* is given by

(40) $$\begin{array}{c} P\left(k\right)=\int d\theta \rho \left(\theta \right)\frac{1}{t}\sum_{t^{\prime }=1}^{t}P\left(k|\theta ,t^{\prime }\right). \end{array}$$

Note that for sufficiently sparse networks where each two connected nodes are typically connected by a link of weight one, the degree of a node can be identified with its strength

(41) $$\begin{array}{c} k_{i}≃s_{i}. \end{array}$$

It follows that in this case the degree distribution can be approximated by the strength distribution and we have that if the hidden variables are power-law distributed with power-law γ (as described in Equation (26)) then also the degree distribution has a power-law tail with the same exponent γ, i.e.,

(42) $$\begin{array}{c} P\left(k\right)=\tilde{C}k^{-\gamma } \end{array}$$

for k≫1.

### 5.6. Random Permutation of the Node Sequence

Here we investigate whether the described network process can be related to the generation of uncorrelated networks. In this way we aim at reconciling the non-equilibrium growing nature of the network model, displaying projectivity, with the properties of exchangeable but not projective uncorrelated network models.

We observe that this expression depends both on the hidden variable and on the time of arrival of the nodes *i* and *j* in the network. However if we consider several realizations of the model in which the times of arrival of node *i* and node *j* are random, but the hidden variables are preserved, we observe that the probability that node *i* and node *j* are connected satisfies

(43) $$\begin{array}{cc} P(a_{ij}=1|\theta_{i},\theta_{j},t=N) & =\frac{1}{N^{2}}\int_{1}^{N}dt_{i}\int_{1}^{N}dt_{j}\int_{1}^{N}d\tau \delta \left(\tau ,\min\left(t_{i},t_{j}\right)\right)\left[1-\exp\left(-\frac{\theta_{i}\theta_{j}}{\langle \theta \rangle \tau }\right)\right]\\ & =\frac{2}{N^{2}}\int_{1}^{N}d\tau \tau \left[1-\exp\left(-\frac{\theta_{i}\theta_{j}}{\langle \theta \rangle \tau }\right)\right]=2\frac{\theta_{i}\theta_{j}}{\langle \theta \rangle N}+o\left(\frac{\theta_{i}\theta_{j}}{\langle \theta \rangle N}\right). \end{array}$$

Therefore if the network is sufficiently sparse, i.e.,

(44) $$\begin{array}{c} \frac{\theta_{i}\theta_{j}}{\langle \theta \rangle N}\ll 1, \end{array}$$

we have that the expected degree $k_{i}\left(\theta_{i}\right)$ of a random node *i* of hidden variable $\theta_{i}$ is given by

(45) $$\begin{array}{c} {\tilde{k}}_{i}\left(\theta_{i}\right)=2\theta_{i}, \end{array}$$

and the probability that a node with hidden variable $\theta_{i}$ is connected with a node with hidden variable $\theta_{j}$ independently of their time of arrival in the network, is given by the uncorrelated network marginal corresponding to the number of nodes in the sample, i.e.,

(46) $$\begin{array}{c} {\tilde{p}}_{ij}=P\left(a_{ij}=1|\theta_{i},\theta_{j},t=N\right)=\frac{{\tilde{k}}_{i}\left(\theta_{i}\right){\tilde{k}}_{j}\left(\theta_{j}\right)}{\langle \tilde{k}\left(\theta \right)\rangle N}. \end{array}$$

Note that in this case if the sample increases in size and includes $N^{\prime }>N$ nodes, the probability that node *i* and node *j* are connected will satisfy

(47) $$\begin{array}{c} {\tilde{p}}_{ij}^{\prime }=P\left(a_{ij}=1|\theta_{i},\theta_{j},t=N^{\prime }\right)=\frac{{\tilde{k}}_{i}\left(\theta_{i}\right){\tilde{k}}_{j}\left(\theta_{j}\right)}{\langle \tilde{k}\left(\theta \right)\rangle N^{\prime }}. \end{array}$$

In this case the network process induces a probability ${\tilde{p}}_{ij}$ that depends on the network size *N* and at the same time enforces the sparseness of the network. In fact the expected degrees $\left\{{\tilde{k}}_{i}\right\}$ of the nodes are only determined the the hidden variable and are independent on the network size.

### 5.7. Entropy of the Network Model

In order to compare our model with hidden variable distribution ρ(θ) to an uncorrelated network ensemble in which the expected degrees are ${\tilde{k}}_{i}=2\theta_{i}$, in this section we use information theory tools. Specifically we will compare the entropy of the two ensembles. The entropy of a network model or of a network ensemble [14,15,16,17,40] is a fundamental tool to evaluate the information content in the network model. It indicates the logarithm of the typical number of networks generated by the ensemble and as such evaluates the complexity of the model and can be used in inference problems [40]. Since for our network model the connection probability $p_{ij}$ of any two pair of nodes is *i* and *j* is given by Equation (31), the entropy of the model is given by

(48) $$\begin{array}{c} S=-\sum_{i<j}\left[p_{ij}\ln p_{ij}+\left(1-p_{ij}\right)\ln\left(1-p_{ij}\right)\right]. \end{array}$$

where in the sparse regime we can approximate $p_{ij}$ with $t_{j}>t_{i}$ as

(49) $$\begin{array}{c} p_{ij}≃\frac{\theta_{i}\theta_{j}}{\langle \theta \rangle t_{j}}. \end{array}$$

Similarly for the uncorrelated network ensemble with connection probability ${\tilde{p}}_{ij}$ the entropy is given by

(50) $$\begin{array}{c} \tilde{S}=-\sum_{i<j}\left[{\tilde{p}}_{ij}\ln{\tilde{p}}_{ij}+\left(1-{\tilde{p}}_{ij}\right)\ln\left(1-{\tilde{p}}_{ij}\right)\right]. \end{array}$$

In order to compare these two entropies we use the explicit expression for the connection probability ${\tilde{p}}_{ij}$ when we put ${\tilde{k}}_{i}\left(\theta_{i}\right)=2\theta_{i}$ which reads

(51) $$\begin{array}{c} {\tilde{p}}_{ij}=2\frac{\theta_{i}\theta_{j}}{\langle \theta \rangle N}. \end{array}$$

By performing a straightforward calculation we find that *S* is given, up to the linear terms in *N*, by

(52) $$\begin{array}{c} S=\langle \theta \rangle \ln(N!)-2N\langle \theta \ln\theta \rangle +N\langle \theta \rangle \ln\langle \theta \rangle +\langle \theta \rangle N \end{array}$$

and that the entropy *S* of our model is smaller than the entropy of the uncorrelated network ensemble. In fact, *S* differs from $\tilde{S}$ only by

(53) $$\begin{array}{c} \Delta S=S-\tilde{S}≃\langle \theta \rangle \ln\left(\frac{N!2^{N}}{N^{N}}\right)≃\langle \theta \rangle N\ln\left(\frac{2}{e}\right). \end{array}$$

The entropy difference ΔS quantifies the information loss when the proposed network process is approximated with its corresponding uncorrelated network model. We observe here that the uncorrelated network model is obtained when the causal construction of the original network model is disregarded and the only retained information is the probability ${\tilde{p}}_{ij}$ that two nodes of hidden variables $\theta_{i}$ and $\theta_{j}$ are connected regardless of their time of arrival in the network. Therefore ΔS captures the loss of information when the causal nature of the original model is disregarded. Interestingly in the large network limit N≫1, |ΔS| is low when compared to *S* revealing the proximity between the two models. Additionally ΔS is only dependent on $\langle \theta \rangle$ indicating that the information loss from one model to the other is independent of the particular distribution of the hidden variables ρ(θ) as long as $\langle \theta \rangle$ is kept constant.

## 6. Statistical Testing of the Model

**Figure 1:**
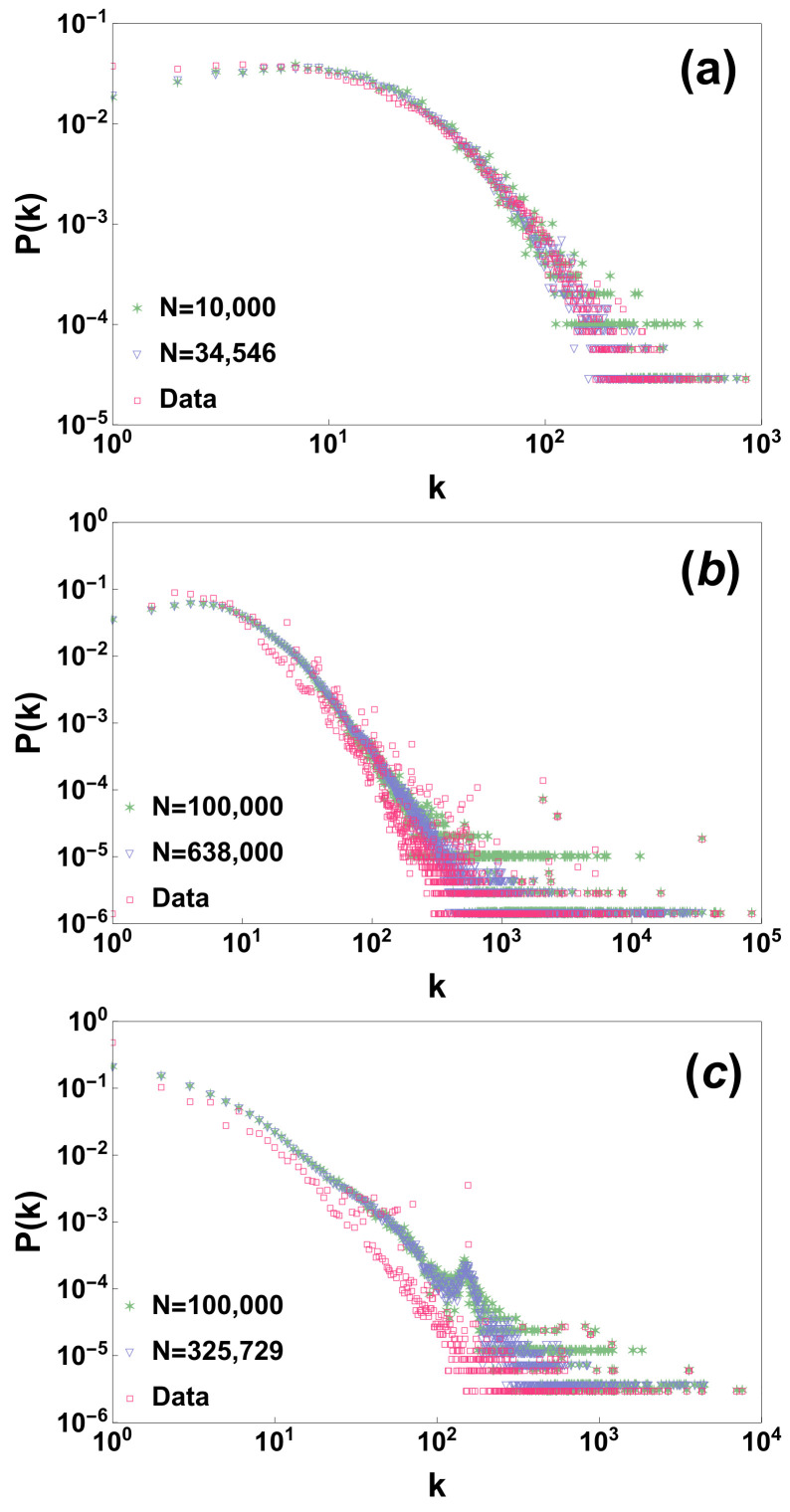
The degree distributions P(k) of the three analysed datasets is compared with the results of the model generated by using all the nodes of the network or with just a subsample of nodes of the network of size *N*. Panels (**a**–**c**) display the results for the arxiv hep-ph citation network [45,46] (N= 34,546) the Berkeley-Stanford web network [47] (N= 685,546) and the Notre Dame web network [48] (N= 325,000) respectively.

In order to study the utility of the proposed model as a null model for sampled data we consider three power-law networks: the arxiv hep-ph (high energy physics phenomenology) citation network [45,46], the Berkeley–Stanford web network [47] and the Notre Dame web network [48] of network sizes N= 34,546, N= 685,230, N= 325,000 respectively. All data are freely available on the Stanford Network Analysis Project webpage. To each node of the network we assign a different label i∈1,2,…,N according to a random permutation of the indices from 1 up to *N*. We then assign to each node *i* of the network a hidden variable

(54) $$\begin{array}{c} \theta_{i}=\frac{1}{2}k_{i}, \end{array}$$

where $k_{i}$ is the observed degree of node *i* in the dataset. Given our random node labelling and the hidden variables ${\left\{\theta_{i}\right\}}_{i\;=\;1,\;2,\;\ldots ,\;N}$ we have generated a random network according to the proposed network process. Interestingly the proposed model preserves to a large extent the degree distribution (see comparison of the real degree distribution with the one generated by the model in Figure 1). Additionally these results are quite stable if we consider a model generated only by adding a subsample of randomly chosen nodes, showing that the model preserves the degree distribution under random sub-sampling of the nodes (see Figure 1).

**Figure 2:**
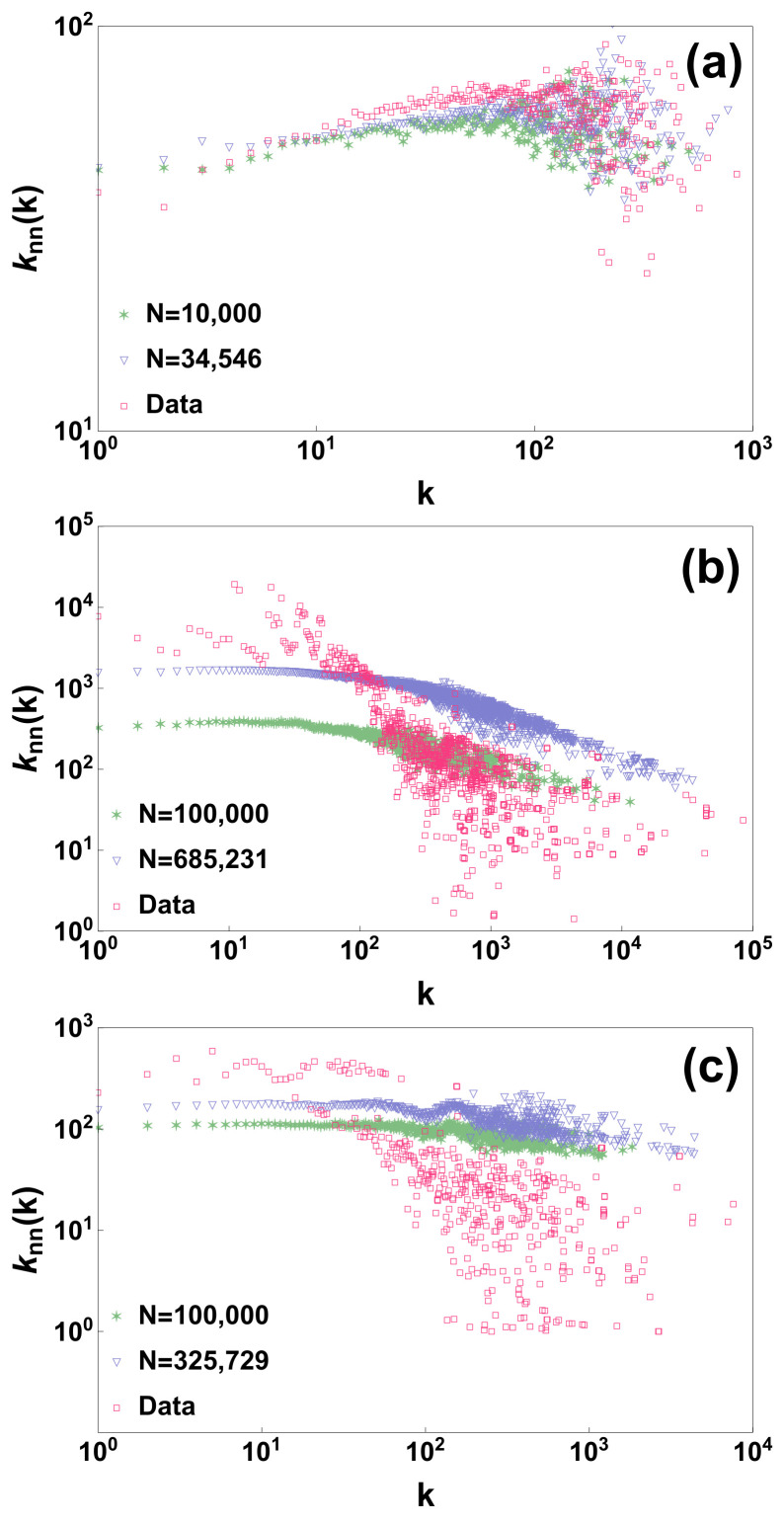
The average degree $k_{nn}\left(k\right)$ of the neighbour of a node of degree *k* of the three analysed datasets is compared with the results of the model generated by using all the nodes of the network or with just a subsample of nodes of the network of size *N*. Panels (**a**–**c**) display the results for the arxiv hep-ph citation network [45,46] (N= 34,546) the Berkeley-Stanford web network [47] (N= 685,546) and the Notre Dame web network [48] (N= 325,000) respectively.

The generated model however is to be considered mostly as uncorrelated. In fact if we compare the degree correlations of the real datasets with the degree correlations of the network generated by the model we observe that the model deviates from the real data and displays very weak/marginal degree correlations (see Figure 2). In fact from the results obtained for the three studied network datasets it seems that the model is able to better reproduce weakly assortative behaviour than strongly disassortative behaviour. In future, modifications of the proposed model could be envisaged to capture also the degree correlations of real datasets.

## 7. Conclusions

In conclusion, we have given a wide overview of the desirability of the projectivity and exchangeability properties in good statistical models and we have emphasized the difficulty in combining these properties with the sparseness of the network. While this problem is a widely discussed subject in statistics of networks and graph theory, here we have proposed a model that provides a trade-off solution. Our model describes a network process in which nodes and links are subsequently added according to a probability dependent on some hidden variables associated to the nodes. As long as the hidden variables are power-law distributed this model generates a scale-free network with the same exponent. This model is projective but not exchangeable. However, the expected probability that two nodes are connected when one considers a random permutation of the sequence in which nodes are added to the network reduces to the expression valid for the marginal of an uncorrelated exchangeable network with the same expected degrees (given by the double of the hidden variables) provided the network is sufficiently sparse. Finally, we tested this model as a statistical null model for scale-free sparse real networks, showing that it can reproduce the degree distribution (but not degree correlations) also if a partial subset of the data is considered.
